# Unusual Perioperative Cardiac Emergency in a Healthy Young Woman

**DOI:** 10.1155/2012/103051

**Published:** 2012-08-16

**Authors:** Pragati Ganjoo, Vijay K. Pandey, Hukum Singh, Monica S. Tandon, Daljit Singh

**Affiliations:** ^1^Department of Anaesthesiology and Intensive Care, GB Pant Hospital, Maulana Azad Medical College, New Delhi 110002, India; ^2^Department of Neurosurgery, GB Pant Hospital, Maulana Azad Medical College, New Delhi 110002, India

## Abstract

Serious cardiac complications occurring during noncardiac surgery in a young and otherwise normal person can be quite alarming for the anesthesiologist. We report here the case of a young, healthy woman who immediately after an uncomplicated spinal surgery developed a clinical picture suggestive of an acute myocardial infarction (MI) with positive relevant investigations. However, she had an abrupt and full clinical recovery and complete normalization of her cardiac investigations within a few days of this event and thereafter continued to lead a normal, symptom-free life unlike the usual course in an MI; her coronary angiography was also normal. A diagnosis of perioperative stress-induced cardiomyopathy or Takotsubo cardiomyopathy was subsequently made. This condition is characterized by a rapid, severe, but reversible, cardiac dysfunction triggered by physical or mental stress. Awareness of this entity should help anesthesiologists manage better this infrequent, but potentially life-threatening, perioperative complication.

## 1. Introduction

Life-threatening cardiac complications during noncardiac surgery are not unexpected in patients at risk for, or with, proven coronary artery disease (CAD). The anesthesiologist usually anticipates and prepares in advance to handle these cases. However, the same occurring in an otherwise normal young adult during a low-risk surgery can be alarming. Acute myocardial infarction (MI) in young adults is well known. Another relatively uncommon condition mimicking an acute MI is Takotsubo cardiomyopathy (TCM) that can present as an unexpected serious perioperative cardiac complication in apparently normal individuals [1–4]. It is characterized by rapid, severe, but reversible, cardiac dysfunction triggered by intense emotional or physical stress, including surgery. Familiarity with this unusual condition is thus important for its appropriate perioperative management. We report here the occurrence of this transient cardiac syndrome in a healthy young woman following an uneventful spinal surgery and briefly discuss the relevant medical literature. 

## 2. Case Report 

A 25-year-old female patient, ASA grade I, underwent laminectomy and excision of an L2-L3 intradural tumor under general anesthesia. Her intraoperative course was largely uneventful and she was awake and hemodynamically stable on transfer to the postoperative area. However, within 30 min, she complained of uneasiness and vomited once. Her blood pressure (BP) decreased to 74/52 mmHg and oxygen saturation to 74%, and her heart rate increased to 128/min. She did not have chest pain, dyspnea, arrhythmias, or cyanosis. Chest auscultation revealed bilateral rales and normal heart sounds. Invasive monitoring was established which revealed a central venous pressure of 10 mmHg, hypoxia, severe metabolic acidosis, and normal electrolytes on arterial blood gas analysis. She was treated with oxygen, soda bicarbonate, furosemide, and dopamine infusion at 7 *μ*g/kg/min. Her ECG showed ST depression in diffuse leads and chest X-ray showed bilateral lung haziness. A 2-D echocardiography revealed regional wall motion abnormalities (RWMA), namely, hypokinesia of basal and mid-septal and mid and apical anterior walls with moderate left ventricular dysfunction (ejection fraction ~30–35%). Troponin-T test done twice, 5 hours apart, was positive (0.16 *μ*g/L and 0.34 *μ*g/L, respectively, normal range at our lab ~0.0–0.1 *μ*g/L). Acute MI was suspected and the patient further treated in the coronary care unit. Percutaneous coronary intervention was considered here but consent for the procedure could not be obtained. She continued to have hypotension (systolic BP ~80–85 mmHg) over the next 2 days despite adequate inotropic support. However, on the 4th postoperative day, her condition improved abruptly and within the next 24 hours she had completely recovered with normal vital parameters and ECG; dopamine was discontinued. Her remaining hospital stay was uneventful and she was discharged after 10 days on beta-adrenergic blocker, angiotensin-converting enzyme inhibitor, and statin therapy and put on regular cardiac followup. Her evaluation after 3 weeks revealed no cardiac manifestations and a normal ECG and echocardiography (no RWMA, ejection fraction ~60%); her cardiac drugs were withdrawn. She subsequently underwent a stress echocardiography which was negative for inducible ischemia. This was followed by a computed tomography (CT) coronary angiography which revealed the coronaries to be normal in origin, caliber, and outline with no evidence of any plaque or stenosis and a normal cardiac function (ejection fraction ~69%, end-diastolic volume ~64.83 mL, end-systolic volume ~44.81 mL, and cardiac output ~4.12 L/min). In her year-long followup until now, she continues to be totally symptom-free and has a normal echocardiography despite resuming all routine work.

## 3. Discussion

The perioperative cardiac events in this patient were completely unexpected. Though her initial presentation was suggestive of an acute MI, her young age and apparently normal prior health made us first think of pulmonary embolism, aspiration, and pneumothorax as the probable causes; these were soon ruled out. Though rare, acute MI is known in young adults [5]. Its causative factors include coronary artery aneurysm and dissection, anomalous origin of coronary arteries, coronary vasospasm due to illicit substances misuse, premature atherosclerotic CAD, and thrombotic occlusion of coronaries in hyper-coagulable conditions like systemic lupus erythematosis, hyperhomocysteinemia, and hormonal contraceptive pill use. Perioperative MI usually occurs during emergence from anesthesia and may be clinically silent, manifesting only as ST-segment depression on ECG and raised troponin levels, as was evident in our patient also. However, the abrupt and complete normalization of her hemodynamic status, ECG, and echocardiography within a few days was inconsistent with an MI where abnormal cardiac changes are expected to persist because of irreversible myocardial damage. This patient's typical clinical picture, highlighted by significant, but short-lived cardiac destabilization followed by full recovery and continued normal symptom-free life, a normal stress echocardiography, and no obvious abnormality seen on CT coronary angiography, is very indicative of TCM, also known as stress-induced cardiomyopathy or broken-heart syndrome [6, 7]. Normalization of cardiac function within days to a few weeks is the cardinal feature of this condition. TCM is often mistakenly diagnosed as an acute MI as both have similar clinical manifestations; 2% of suspected MI patients were subsequently seen to have TCM [8]. The modified Mayo Clinic criteria for diagnosing TCM include (a) transient hypokinesis, dyskinesis, or akinesis of the left ventricular mid-segments with or without apical involvement, the RWMA extending beyond a single epicardial vascular distribution, and presence of a stressful trigger often, but not always; (b) absence of angiographic evidence of obstructive CAD or of acute plaque rupture; (c) new ECG abnormalities (ST segment and/or T-wave changes) or modest elevation in cardiac troponin level; (d) absence of pheochromocytoma, myocarditis, or hypertrophic cardiomyopathy [9]. Several variant forms of TCM with different patterns of clinical presentation and cardiac wall motion abnormalities have also been reported [10]. Though more commonly seen in postmenopausal women aged 62–75 years, TCM is also reported in younger patients [6, 10]. Unlike CAD, it has no known risk factors like hyperlipidemia, smoking, positive family history, hypertension, and diabetes. Some of the proposed etiologies for TCM include excess catecholamine-induced myocardial stunning, multivessel coronary artery spasm, transient epicardial coronary thrombosis, impaired cardiac microvascular function, and dynamic left ventricular outflow tract obstruction. The initial management of TCM is similar to that of an MI. Close cardiac followup and serial echocardiograms are recommended till complete cardiac recovery and beta-adrenergic blocker therapy may be beneficial [6, 7]. Though TCM has an excellent prognosis, life-threatening complications like congestive cardiac failure (44–57% of cases), cardiogenic shock (15–45%), pulmonary edema, dysrhythmias, thromboembolism, left ventricular wall rupture, and death have been reported [8, 10].

Although infrequently encountered, perioperative TCM is a well-established clinical syndrome reported in a variety of surgeries, particularly following emergence from anesthesia [1–4]. For anesthesiologists, however, many vital issues regarding TCM still remain unresolved like preoperative identification of susceptible patients and high-risk surgeries, identifying the various triggers, the best anesthesia technique to help prevent onset, and the optimal therapy in the acute phase. To understand these concerns better, large population cohort studies and perioperative patient registries have been suggested [10].

This case highlights the importance of TCM as a potential cause of severe perioperative hemodynamic instability and emphasizes the need for adequate preparedness to manage serious cardiac complications in all surgical patients, irrespective of their age and preoperative state. More frequent diagnosis and improved management of perioperative TCM are likely with its increased awareness and reporting among anesthesiologists. 
